# An Insight Into COVID-19: A 21st Century Disaster and Its Relation to Immunocompetence and Food Antioxidants

**DOI:** 10.3389/fvets.2020.586637

**Published:** 2021-01-13

**Authors:** Faisal Siddique, Rao Zahid Abbas, Muhammad Khalid Mansoor, Etab Saleh Alghamdi, Muhammad Saeed, Muhammad Mazhar Ayaz, Moazur Rahman, Muhammad Shahid Mahmood, Asif Iqbal, Maida Manzoor, Asghar Abbas, Asif Javaid, Irshad Hussain

**Affiliations:** ^1^Department of Microbiology, Cholistan University of Veterinary and Animal Sciences, Bahawalpur, Pakistan; ^2^Department of Parasitology, University of Agriculture, Faisalabad, Pakistan; ^3^Department of Microbiology, The Islamia University of Bahawalpur, Bahawalpur, Pakistan; ^4^Department of Food and Nutrition, Faculty of Human Sciences and Design, King Abdul-Aziz University, Jeddah, Saudi Arabia; ^5^Department of Poultry Sciences, Cholistan University of Veterinary and Animal Sciences, Bahawalpur, Pakistan; ^6^Department of Parasitology, Cholistan University of Veterinary and Animal Sciences, Bahawalpur, Pakistan; ^7^School of Biological Sciences, University of the Punjab, Lahore, Pakistan; ^8^Institute of Microbiology, University of Agriculture, Faisalabad, Pakistan; ^9^Department of Parasitology, Riphah International University, Lahore, Pakistan; ^10^Department of Veterinary and Animal Sciences, Muhammad Nawaz Shareef University of Agriculture, Multan, Pakistan; ^11^Department of Animal Nutrition, Cholistan University of Veterinary and Animal Sciences, Bahawalpur, Pakistan; ^12^Department of Microbiology, University of Veterinary and Animal Sciences, Lahore, Pakistan

**Keywords:** COVID-19, genome, viral pneumonia, health emergency, ACE2, immune-competence, antioxidants

## Abstract

Coronavirus Disease 2019 (COVID-19) ranks third in terms of fatal coronavirus diseases threatening public health, coming after SARS-CoV (severe acute respiratory syndrome coronavirus), and MERS-CoV (Middle East respiratory syndrome coronavirus). SARS-CoV-2 (severe acute respiratory syndrome coronavirus type 2) causes COVID-19. On January 30, 2020, the World Health Organization (WHO) announced that the current outbreak of COVID-19 is the sixth global health emergency. As of December 3, 2020, 64 million people worldwide have been affected by this malaise, and the global economy has experienced a loss of more than $1 trillion. SARS-CoV-2 is a positive-sense single-stranded RNA virus belonging to the *Betacoronavirus* genus. The high nucleotide sequence identity of SARS-CoV-2 with the BatCoV RaTG13 genome has indicated that bats could be the possible host of SARS-CoV-2. SARS-CoV-2 penetrates the host cell via binding its spike protein to the angiotensin-converting enzyme 2 (ACE2) receptor, which is similar to the mechanisms of SARS-CoV and MERS-CoV. COVID-19 can spread from person to person via respiratory droplets and airborne and contaminated fomites. Moreover, it poses a significant risk to smokers, the elderly, immunocompromised people, and those with preexisting comorbidities. Two main approaches are used to control viral infections, namely, vaccination, and biosecurity. Studies to analyze the antigenicity and immunogenicity of SARS-CoV-2 vaccine candidates are underway, and few vaccines may be available in the near future. In the current situation, the Human Biosecurity Emergency (HBE) may be the only way to cope effectively with the novel SARS-CoV-2 strain. Here, we summarize current knowledge on the origin of COVID-19 as well as its epidemiological relationship with humans and animals, genomic resemblance, immunopathogenesis, clinical-laboratory signs, diagnosis, control and prevention, and treatment. Moreover, we discuss the interventional effects of various nutrients on COVID-19 in detail. However, multiple possibilities are explored to fight COVID-19, and the greatest efforts targeted toward finding an effective vaccine in the near future. Furthermore, antioxidants, polyphenols, and flavonoids, both synthetic and natural, could play a crucial role in the fight against COVID-19.

## Introduction

Coronaviruses are emerging as a threat to people in the 21st century. They are responsible for high morbidity and mortality rates, especially in the elderly and immunocompromised people. In the last two decades, two human coronaviruses (HCoVs) have been reported to cause mild disease (1). In 2003, SARS-CoV (severe acute respiratory syndrome coronavirus) triggered the first human outbreak in Guangdong Province, China. It affected more than 20 countries worldwide due to international travel (2). A total of 8,098 cases were registered, of which ~916 died during the 2002–2003 SARS-CoV wave (3). At this time, SARS-CoV was the emerging public health infectious outbreak of this century. However, the pandemic ended in 2004, but the estimated global economic loss exceeded $90 billion (4).

After a decade of SARS-CoV infection, the emergence of a new type of coronavirus called MERS-CoV (Middle East respiratory syndrome coronavirus) has challenged the world. Since November 2019, MERS-CoV has affected 2,494 people worldwide, with ~858 deaths (5). Based on molecular and phylogenetic studies, the bats were identified as a natural host of SARS-CoV and MERS-CoV (6).

On 31 December 2019, the third pandemic of a new variant of *Betacoronavirus* occurred in a seafood market in Wuhan, Hubei, China (7). The three new coronavirus epidemics first emerged among the Chinese population, and this is probably due to their traditional eating habits of wild animals such as bats, snakes, foxes, and dogs (8). At the end of February 2020, this novel viral strain was named 2019-nCoV and its disease COVID-19 by the World Health Organization (WHO). The International Committee on Taxonomy of Viruses (ICTV) has declared that the virus has been classified as SARS-CoV-2 (9, 10).

This catastrophe was linked to vast markets of animals and seafood, and research continues to establish the origins of this infectious disease. The primary investigation found that SARS-CoV-2 has a close homology to the amino acid sequence of SARS-CoV and binds to the same receptor (ACE2) as SARS-CoV and MERS-CoV. To date, more than 200 countries/regions have been affected by the COVID-19 pandemic. As of December 3, 2020, ~64 million confirmed cases had been reported, including 1.5 million worldwide deaths due to the COVID-19 pandemic.

On January 30, 2020, the WHO declared that the COVID-19 pandemic was the sixth global public health emergency, preceded by influenza (H1N1) in 2009; Ebola hemorrhagic fever in West Africa in 2014 and 2016; poliomyelitis in 2014; Zika virus outbreak in 2016; and Ebola epidemics in the Democratic Republic of the Congo in 2019 (11, 12). The disease is spread to healthy individuals *via* respiratory droplets, contaminated mites, and airborne methods. Furthermore, cooperation among people employed in the health sector, physicians, medical personnel, and private and government institutions is required to stop its spread around the world (13).

## Epidemiology OF SARS-CoV-2

At the end of December 2019, the first case of COVID-19 was reported in Wuhan, China. The COVID-19 pandemic has changed people's habits and lives worldwide, and this includes restrictions on travel and tourism. Many countries have implemented a policy of lockdown to limit movements of citizens from one region to another within the country (4).

Preliminary analyses have shown that the origin of the new virus strain is linked to the Chinese market for seafood. SARS-CoV-2 crossed the species boundary and was transmitted from animals to humans during November or December 2019, and clinical cases were observed almost at the end of December (14). Chinese researchers have rapidly isolated SARS-CoV-2 from a patient with pneumonia. This new virus's genome sequence is close to that of SARS-CoV (80%) and bats (96%). Subsequently, several studies have shown that the bat could be the potential host reservoir of SARS-CoV-2, although bats are not sold in this seafood market (15, 16). To date, there is no definitive phylogenetic proof that SARS-CoV-2 has appeared in the Chinese seafood market (17).

Based on the phylogenetic and proteomic analysis, potential intermediate hosts of SARS-CoV-2 include snakes, turtles, and pangolins (18). SARS-CoV-2 is primarily transmitted to humans through respiratory droplets of people infected with SARS-CoV-2 while coughing, sneezing, and talking. These heavy water droplets are suspended in the atmosphere and rapidly fall to the ground or onto solid objects. If people touch the hands, mouths, or tainted clothes or shoes of an infected person, they are more likely to catch COVID-19 (12).

Moreover, the transmission of SARS-CoV-2 occurs through airborne droplets in an indoor environment, extended access to respiratory debris, and improper ventilation. Airborne spread is distinguished from droplet spread based on the size of expired droplets of the infected person: if the particle size is >5 μm, it is a droplet; if the particle size is <5 μm, it is an aerosol. The SARS-CoV-2 reside within droplet nuclei. SARS-CoV-2 is stable and viable in aerosols for several hours compared to SARS-CoV and can be spread over distances >1 m to healthy individuals. Few research reports have documented the airborne transmission of COVID-19, which might cause a possible aerosolized spread of the disease (19–21). Airborne spread of SARS-CoV-2 has been observed in cats and ferrets. Moreover, a close relationship between air pollutants, for example, PM_2.5_, PM_10_, NO_2_, and O, and COVID-19 has been observed. SARS-CoV-2 can bind to atmospheric contaminants and can spread through the air. Several studies have shown promising results in isolating the SARS-CoV-2 genome in airborne samples using a polymerase chain reaction assay (22, 23). The epidemiological comparison of different biological features among various important coronaviruses (SARS-CoV, MERS-CoV, and SARS-CoV-2) is demonstrated in Table 1.

**Table 1:** Epidemiological comparison of human coronaviruses.

**Biological features**	**SARS-CoV-1**	**MERS-CoV**	**SARS-CoV-2**
Classification	β -CoV (lineage B)	β-CoV (lineage C)	β-CoV (lineage B)
First outbreak	2002, Guangdong, China	2012, Saudi Arabia	2019, Wuhan, China
Countries affected	32	109	210
Estimated Ro value	2	<1	2.68
Virus spreader	Bats	Bats	Bats
Intermediate host	Palm civets, raccoons, and dogs	Camels	Pangolins and minks
Incubation period	4–7 days	2–15 days	4–14 days
Transmission	Respiratory droplets	Respiratory droplets	Respiratory droplets
Season	Winter	Winter	Winter
Receptors to host cells	hACE2	hACE2	hACE2
Signs and symptoms	Fever, headache, dry cough, dyspnea, diarrhea, and ARDS	Fever, chills, sore throat, dyspnea, diarrhea and vomiting, acute renal impairment, and ARDS	Fatigue, fever, dry cough, dyspnea, anosmia, tachypnea, and ARDS
Mortality (%)	9%	34.5%	3.6%
References	(24–27)	(4, 7, 9, 28–30)	(12, 15, 18, 23, 31, 32)

*SARS-CoV-1, severe acute respiratory syndrome coronavirus; MERS-CoV, Middle East respiratory syndrome coronavirus; SARS-CoV-2, severe acute respiratory syndrome coronavirus type 2; hACE2, human angiotensin-converting enzyme 2; ARDS, acute respiratory distress syndrome*.

Smokers, people aged >60 years, doctors, paramedical staff, immunocompromised patients, children (<5 years old), patients with chronic lung and heart diseases, and family members (i.e., relatives, mother, father, wife, and children) and friends of infected patients are at significant risk of COVID-19 (33).

Evidence from the global epidemic suggests that comorbidities, such as hypertension, diabetes, and obesity, are the leading causes of death in patients with COVID-19 (34, 35). Hypertension is the highest preexisting comorbidity among patients with COVID-19 and is the leading cause of death (36). Patients with hypertension are usually treated with renin-angiotensin system inhibitors (ACEIs) and angiotensin receptor blockers (ARBs). Since ACEIs and ARBs can significantly increase ACE2 expression, several researchers speculate that they are correlated with an increased risk of COVID-19. Moreover, renin-angiotensin inhibitors are commonly used to treat patients with cardiovascular diseases who have an increased mortality risk during COVID-19 epidemics (31). Diabetes is the third most common potential comorbidity among patients with COVID-19. Individuals with diabetes are more vulnerable to diseases and are at high risk of developing multiple comorbidities, such as cardiovascular diseases and hypertension (37). In addition, the critical risk factors for COVID-19 infection are preexisting respiratory conditions, such as asthma and chronic obstructive pulmonary disease, and chronic liver disease (35). The average incubation period of SARS-CoV-2 is 4–14 days (38).

## Epidemiological Relationship Between COVID-19 and Animals

The Coronaviridae family affects various types of animals, such as poultry, goats, dogs, cats, horses, and fish, and human beings (39). In most cases, their infections are subclinical or asymptomatic (40). However, enteritis has been observed in cattle and horses; respiratory illness has been seen in dogs, cats, and poultry; and asymptomatic to symptomatic respiratory infections have been detected in humans (32, 41). Coronaviruses induce clinical illness in laboratory animals such as mice, rabbits, rats, and guinea pigs. Among the coronaviruses, bovine coronaviruses, SARS-CoV, MERS-CoV, HCoV-NL63, and SARS-CoV-2 have zoonotic origins (42). Zoonotic coronaviruses, especially SARS-CoV, MERS-CoV, and SARS-CoV-2, can cross the species barrier due to genetic recombination in the receptor-binding domain (43). COVID-19 has spread worldwide due to the instability of the RNA replicase enzyme, which is incapable of proofreading activity, and an increased mutation rate in the receptor-binding domain of the spike protein gene (35). The genetic reshuffling of the spike protein gene can increase the spread, virulence, and host species from animals to humans (44).

Preliminary research has shown that the origin of the first case of COVID-19 was observed in the seafood market in Wuhan, Hubei, China. Restaurants in this market offer all sorts of wild animal meat, such as broilers, snakes, tortoises, bats, pangolins, marmots, and hedgehogs (24). The initial thought was that the SARS-CoV-2 virus had been transmitted from animals to humans through the seafood industry. This hypothesis was reinforced by the previous zoonotic coronaviruses such as SARS-CoV and MERS-CoV (45). MERS-CoV was transmitted through nasal secretions, feces, and milk (46). The literature has shown that certain bat-origin coronaviruses, such as SARS-CoV and MERS-CoV, have infected humans. As a result, scientists predicted the role of bats in the emergence and circulation of the recent COVID-19 epidemic (47, 48). Recently, a Chinese researcher reported that the genomic structure of SARS-CoV-2 was identical to that of SARS-CoV (80%) and horseshoe bat (96%) and indicated that the bat may be the potential host reservoir of SARS-CoV-2. Hence, bats are responsible for transmitting zoonotic diseases to humans (49). The spike protein of SARS-CoV-2 is similar to that of the pangolin coronavirus. Therefore, bats and pangolins are considered to be sources of SARS-CoV-2 in humans (25).

To date, two cases of COVID-19 have been reported in two separate breeds of dogs, that is, Pomeranian, and German Shepherd (50). A Pomeranian dog was regarded to be a possible intermediate host due to multiple swaps in the dog's ACE2 receptor after its owner was found to be COVID-19 positive, indicating that SARS-CoV-2 had crossed the species barrier (17). In Hong Kong, two German shepherd dogs were also found to be positive for COVID-19 out of 15 dogs tested. The genomic sequences of SARS-CoV-2 isolated from both dogs were similar to those found in human cases, suggesting human-to-dog transmission (28).

The first case of cat infection with COVID-19 was registered at the end of March 2019. The history of these infections in dogs and cats has indicated that their owners have been infected with COVID-19. They both developed immunoglobulins that fight infection. These findings may suggest human-to-animal transmission of COVID-19 (29). Other countries such as Belgium, France, Germany, Russia, and the United States have also documented cases of COVID-19 in cats. Based on these observations and other laboratory research, it can be concluded that these two companion animals are prone to SARS-CoV-2 (30). Cats are sensitive and can spread the disease to other naive cats, whereas dogs are less resistant to infection (23). Laboratory animals, particularly golden Syrian hamsters, have been infected with SARS-CoV-2. High viral loads in the upper and lower respiratory tracts were observed within the first week of infection (51). Moreover, ferrets are highly susceptible to SARS-CoV-2 and effectively transmit SARS-CoV-2 to other naive ferrets via direct, indirect, and airborne transmission. SARS-CoV-2 replicates in the upper respiratory tract of ferrets, making them an acceptable candidate animal model for the evaluation of antiviral drugs or vaccine candidates for COVID-19 (52).

The SARS-CoV-2 outbreak has been reported in four mink farms in the Netherlands. Histopathological results showed that minks died of pneumonia caused by SARS-CoV-2, verified by reverse transcription-polymerase chain reaction (RT-PCR) examination. However, animal transmission and minimal accidental spillover from humans have been observed. The genomic sequence cluster of SARS-CoV-2 was similar to that of minks with seven nucleotide variations. The molecular findings have confirmed its zoonotic importance (53).

Domestic animals like pigs, camels, goats, donkeys, horses, sheep, and poultry are not found to be receptive to SARS-CoV-2 (22, 23). Wild animals like tigers, lions, Egyptian fruit bats, African green monkeys, and rhesus monkeys have been reported to be susceptible to COVID-19 (42). A recent study has shown that Nadia, a 4-year-old tiger, exhibited respiratory symptoms such as coughing and sneezing on March 27, 2020, in the Bronx Zoo in New York City. Samples were obtained and a COVID-19 positive test was found (54). Later on, no confirmatory evidence was obtained suggesting that canines can acquire COVID-19 disease or transfer the virus to humans (55).

## Genomic Categorization of Coronaviruses (COVs)

Coronaviruses are a set of viruses that cause infections in humans and feathered animals. According to ICTV, coronaviruses belong to the Riboviria realm, Nidovirales order, Coronaviridae family, Orthocoronavirinae subfamily, and *Betacoronavirus* genus (56). They are comprised of an encapsulated lipid bilayer and a non-segmented RNA genome. They are the largest viruses among RNA viruses, ~34 kb in size (57). The electron micrograph of the coronavirus has a spike-like or stick-like surface protein close to the crown. The word “corona” is derived from the Latin word “*corona*,” meaning crown (58).

Coronaviruses are classified into four genera, alpha, beta, gamma, and delta coronaviruses, based on their protein sequences. Most human coronaviruses belong to the *Betacoronavirus* genus that is further subcategorized into lineages A, B, C, and D (59). Bats and rodents serve as intermediate hosts for alpha- and betacoronaviruses, whereas birds act as intermediate hosts for gamma- and deltacoronaviruses. Coronaviruses have crossed their biological limits and have emerged as new human pathogens, such as SARS-CoV-2. To date, six Betacoronaviruses have been isolated and identified globally as follows: HCoV-HKU1, HCoV-OC43, SARS-CoV, MERS-CoV, HCoV-229E, and SARS-CoV-2 (60).

High mortality rates have been seen in zoonotic SARS-CoV and MERS epidemics over the last two decades, whereas other coronavirus classifications are typically associated with negligible upper respiratory tract disease. However, they can lead to complex diseases, if they affected an immunocompromised person (26).

The genetic sequence of SARS-CoV-2 belongs to the lineage B betacoronaviruses that is similar to more than 90% of the bat and pangolin coronavirus genomes. Bats are suspected to be the natural carriers of the novel coronavirus COVID-19 based on complete virus genome sequences and phylogenetic analysis. SARS-CoV-2 is known to use the same receptors as SARS-CoV, such as angiotensin-converting enzyme 2, to infect humans (61). The SARS-CoV-2 genetic material serves as an infectious agent containing a 5′-methylated cap and a 3′-polyadenylated tail. The complete genome sequence of SARS-CoV-2 contains 29,811 nucleotides consisting of 8,903, 5,482, 5,852, and 9,574 adenosines, cytosines, guanines, and thymines, respectively (27).

SARS-CoV-2 consists of different structural proteins, including spike (S), membrane (M), envelope (E), and nucleoproteins (N). Petal or club-shaped S proteins are tightly packed membrane glycoproteins that help bind viruses to host cells (62, 63). The pentameric small-integer E protein is involved in the assembly and pathogenesis of viruses (64). The integral M protein of type III is connected to the matrix formation. The N protein leads to the encapsidation of the genome, protein, and RNA synthesis. All these structural proteins (S, M, E, and N) of SARS-CoV-2 can act as antigens to human cells, stimulate the development of antibodies, and increase the immune response of T cells (16).

## SARS-CoV-2 Pathogenesis

SARS-CoV-2 virulence begins after the viral entry through inhalation of respiratory and airborne droplets or contact with infected fomites. The pathogenesis of SARS-CoV-2 depends on various factors, such as age, sex, preexisting comorbidities, immune status, nutritional status, and smoking. Human respiratory epithelial cells contain receptors called hACE2 (human angiotensin-converting enzyme 2) (65). This enzyme lowers blood pressure by converting angiotensin II to angiotensin 1–7. Moreover, other human coronaviruses, e.g., SARS-CoV, MERS-CoV, and HCoV-NL63, bind to ACE2 receptors on host cells (66).

The SARS-CoV-2 S protein is bound to the hACE2 receptor of the host cells. This attachment is required for the initiation of pathogenesis (67, 68). S protein is further subdivided into two active subunits, S1 and S2. A similar pattern of S protein subdivision has been reported for MERS-CoV infection (69, 70). The replication of the SARS-CoV-2 genome occurred in the cytoplasm. The virus produces various structural and non-structural proteins using the host cell's machinery. Infectious viruses are released through host cell lysis (71).

## Immune Response of Host vs. SARS-CoV-2

The SARS-CoV-2 antigen is recognized by different antigens-presenting cells, for example, alveolar macrophages and monocytes that patrol the epithelial cells of the respiratory tract. Virus-specific cytotoxic T lymphocytes recognize these antigenic peptides by means of MHC (major histocompatibility complex) molecules. Pro-inflammatory cytokines, e.g., IL-10, IL-2, TNF-α, IL-10, G-CSF, and MCP-1, were observed during infection with SARS-CoV-2 (72).

The high level of these inflammatory cytokines is known as the cytokine storm, and it has an essential role in the determination of SARS-CoV-2 virulence. Similar findings were documented during SARS-CoV and MERS-CoV infections (73).

This cytokine storm can lead to life-threatening complications, such as developing hypoxic conditions, ARDS (acute respiratory distress syndrome), breathlessness, pneumonia, heart failure, and eventually death. ARDS is a common cause of death in SARS-CoV, MERS-CoV, and SARS-CoV-2 infections. However, little information is available about cytokine storms, and the detailed immune mechanism of SARS-CoV-2 infection is still unknown (74).

Antigen-presenting cells also activate virus-specific B and T cells. The concentration of CD^4+^ and CD^8+^ T has decreased significantly in the peripheral blood of infected individuals with COVID-19. Similar findings have been documented in SARS-CoV and MERS-CoV infections (75). Typical patterns of immunoglobulin development, e.g., IgM and IgG, have been observed during acute SARS-CoV infections. Immunoglobulin production starts after 3 days of infection and reaches an optimum level on day 14 (76). These findings can provide useful evidence for vaccine preparation to cure SARS-CoV-2. Coronaviruses, particularly SARS-CoV and MERS-CoV, use a number of mechanisms to modulate host immune response, including the development of double-membrane vesicles that inhibit INF-I pathways and affect different antigen-presenting cells (77).

## Clinical-Laboratory Cautions of SARS-CoV-2

Clinical signs of COVID-19 include respiratory tract, digestive tract, and generalized symptoms of the body. The clinical indication of COVID-19 disease occurs 6 days after incubation. The most common signs and symptoms of COVID-19 infection are as follows: fever, 99%; dyspnea, 80%; fatigue, 70%; dry cough and anosmia, 59.4% (67, 78). No clinical signs were seen in neonates (78); however, a newborn was tested positive for SARS-CoV-2 at Tongji Hospital, Wuhan, China. Neonates can be infected through perinatal or uterine pathways (79). Asymptomatic carriers have been found among individuals infected with SARS-CoV-2 and clinical symptoms such as cough, fever, and fatigue have not been observed. These carriers have the capacity to shed the virus for up to 21 days and are a possible source of disease transmission to healthy individuals (4). Serious signs and symptoms of COVID-19 are primarily seen in elderly people with cardiovascular disease, hypertension, asthma, and diabetes and those with compromised immune function (80). The SARS-CoV-2 RNA was isolated from stool samples in patients, suggesting that it may be transmitted via the fecal-oral pathway if the virus was intact (81).

The clinical signs and symptoms of COVID-19 contagion are classified based on the severity of infection, which would depend on various risk factors such as age, smoking habit, concurrent bacterial and viral infections, and immune status. The first category is mild illness (81%): patients experience mild symptoms, such as slight fever, nasal congestion, cough, headache, rhinorrhea, nausea, vomiting, muscle pain, diarrhea, abdominal pain, sore throat, loss of taste, and smell; however, they do not suffer from shortness of breath or trouble breathing and have a normal radiographic image (76). Mild diseases are typically managed outside the hospital (82). The second category is a moderate form of the disease in which patients show respiratory symptoms of dyspnea, dry cough, wheezing, and tachypnea (RR >30/min) (83). The third category is the severe form of the disease in which patients presented with sepsis, kidney injury, liver damage, intense pneumonia, ARDS, respiratory rate >30/min, PaO_2_/FiO_2_ < 300, and SpO_2_ ≤ 93%, with a moderate or absent fever (79, 82). The severe or critical form of COVID-19 causes ARDS that could be responsible for worsening respiratory failure. Two forms of ARDS are clinically identified in patients based on PaO_2_/FiO_2_ values as follows: moderate form, PaO_2_/FiO_2_ values = 100 mmHg−200 mmHg; extreme form, PaO_2_/FiO_2_ values ≤ 100 mmHg. Moreover, ARDS has been diagnosed with computed tomography (CT) scanning in which we studied crazy paving, consolidation, ground-glass opacity, and peripheral disease distribution (84).

Currently, ~15% of people require hospitalization (usually for mild to extreme pneumonia), whereas the other 5% have serious illness and need further help. Patients with COVID-19 with preexisting comorbidities have higher mortality rates. These comorbidities include cardiovascular diseases, hypertension, diabetes, and tumor problems. Lower mortality rates have been observed in patients with no comorbidities (35, 36). Laboratory observations of COVID-19 infection detected increased ALT (alanine transaminase), creatine kinase, lactate dehydrogenase, C-reactive protein, plasma interleukins (IL-7, TNFa, IL-2, IL-10, and MCPI), erythrocyte sedimentation rate, blood urea level, and decreased CD4 and CD8 cells (85, 86).

## Diagnosis of SARS-CoV-2

The confirmatory diagnosis of COVID-19 is primarily dependent on history (traveling, age, concurrent infection, etc.), signs and symptoms, and laboratory diagnostic techniques (87). Two types of laboratory diagnostic techniques are used in the diagnosis of SARS-CoV-2. The first is a molecular diagnostic technique, which can recognize part of the viral genome in respiratory specimens. The second is serology or antibody testing, which can identify unique SARS-CoV-2 antibodies in serum samples. Symptomatic or asymptomatic carriers can be identified by molecular tests, as these tests are highly sensitive in order to avoid false-negative results. On the other hand, serological tests can detect the SARS-CoV-2 antigen in patients, as they are specific to the exclusion of false-positive findings (88).

According to WHO, throat swab, nasopharyngeal swab, and samples of sputum, urine, and serum should be collected from the suspected person. If a person had a positive test result, the test should be repeated one more time to double-check. A negative result with robust clinical manifestation should also be repeated (85). Samples such as nasopharyngeal swabs and sputum were collected from infected patients for virus isolation (87). Moreover, the samples have been inoculated into the kidney fibroblasts (Vero cells) of the African green monkey. The electron micrograph of the supernatant has verified the existence of SARS-CoV-2. Real-time RT-PCR is commonly used for an early diagnosis of atypical pneumonia caused by SARS-CoV-2. The commercially available RT-PCR diagnostics for COVID-19 are either one-step or two-step RT-PCR techniques. The main features of the One-Step RT-PCR assay are high detection speed, minimal cross-contamination, being reproducible, low human error rate. On the other hand, the two-step RT-PCR technique is versatile and tunable with high sensitivity and low detection (89, 90).

Many commercially available RT-PCR-based kits have been used worldwide for COVID-19 as follows: Abbott RealTime SARS-CoV-2, Roche's cobas® SARS-CoV-2 Test, Cepheid's Xpert® Xpress SARS-CoV-2, DiaSorin's Simplexa™ COVID-19 Direct RT-PCR kit, and Hologic's Panther Fusion® SARS-CoV-2 (91). Some alternative molecular diagnostic tools, such as LAMP and CRISPR, are used to diagnose COVID-19. The FDA approved the Atila BioSystems iAMP COVID-19 Detection Kit and the Sherlock™ CRISPR SARS-CoV-2 kit for the LAMP and CRISPR tests, respectively. Both kits showed a 100% sensitivity (89). In addition, the full genome sequence and phylogenetic analysis of the virus isolate showed that the isolate had more than 99.9% sequencing identification with other publicly available SARS-CoV-2 genomes (92). Lateral flow immunoassay (LFIA) and enzyme-linked immunosorbent assay (ELISA) are two serologically important tests widely used for the diagnosis of COVID-19 (90). IgM and IgG antibodies are identified by various serological tests. Serological tests can offer false-negative and false-positive results due to cross-contamination of antibodies to other human coronaviruses such as SARS-CoV, MERS-CoV, HKU1, 229E, and NL63. LFIAs are a simple, quick (5–20 min), long-use (up to 18 months), and relatively inexpensive method for detecting COVID-19 antibodies. However, some drawbacks of these tests have been found in positive COVID-19 cases, such as an increased risk of an operator getting infected and false-negative outcomes (93). In many countries/regions, including Spain, Taiwan, Switzerland, Denmark, China, France, Brazil, the United States, South Korea, Indiana, Pakistan, and Malaysia, seroprevalence rates have been reported in COVID-19 hot spots. Among them, Spain has the highest prevalence rate, followed by other countries. However, over time, the rate may increase or decrease. Periodic surveillance of seroprevalence at each site should be determined to ascertain the epidemiology of COVID-19 (94).

## Control and Prevention of SARS-CoV-2

COVID-19 is an infectious disease that is spread from person to person. Two approaches are used to monitor the infection of COVID-19, namely, Human Biosecurity Emergency (HBE) and vaccination (95). HBE can be used to monitor the latest strain on a global scale. HBE is declared to lock a specific infected area by preventing the movement of any person or object. To date, the city of COVID-19 birth, Wuhan, China, successfully combated the infection after a 76-days complete lockdown. During lockdown, schools, colleges, universities, public and private transport, malls, grocery shops, and borders were closed and people were confined to their homes (96). For the general public, the risk of infection can be minimized by the use of Infection Prevention and Control (IPC) strategies, such as the use of face masks, washing hands with soap and sanitizer for 20 s, maintaining an acceptable social distance (1 m or 3 feet), and self-isolation if you feel sick (19). People with low immunity are advised to avoid group gatherings, close contact with wild and domestic birds and animals, and rubbing their noses, mouths, and eyes with dirty hands (39). Doctors and paramedical staff must wear FFP3 masks or N95 masks, eye goggles, medical gloves, and protective gowns during the care of infected persons (97, 98).

Till now, no specific antiviral drug or vaccine is available to treat COVID-19 (99). Strict quarantine and various therapeutic interventions help control COVID-19 globally, such as the use of convalescent plasma, antiviral drugs, monoclonal antibodies, herbal medicine (mAbs), neutralizing antibodies, antibiotics, nutritional supplements, fluid management, oxygen therapy, and intravenous immunoglobulins (100–103).

Convalescent plasma obtained from patients recovering from COVID-19 provides rapid immunity to infected individuals. This technique offers optimal healing outcomes and can be used as a prophylaxis in high-risk immunocompromised patients and healthcare staff such as nurses, physicians, and lab technicians. Previously, this approach was used worldwide for the SARS-CoV and MERS-CoV epidemics (104). Convalescent plasma with monoclonal antibodies is now commercially available. However, passive immunity via convalescent plasma has resulted in rapid, short-term immunity in infected patients. Therefore, vaccine production is a prerequisite for long-term and stable immunity for infected individuals. Maximum protective results were achieved on day 5 of COVID-19 infection following the use of convalescent plasma. The literature revealed that patients weighing 50–80 kg were given two plasma units (200–250 mL each). However, in extreme cases, a dose of 500 ml was injected intravenously after 12 h (105, 106). Monoclonal antibodies, given intravenously, may have a crucial role in the prevention and control of COVID-19. Furthermore, monoclonal antibodies reduced inflammation by inhibiting FcR stimulation. Nevertheless, these therapies have some vulnerabilities such as abnormal reactions, short-term safety, toxins, and dissemination of causative agents (107, 108).

Traditional Chinese Medicine (TCM) plays an integral role in reducing COVID-19 symptoms through various mechanisms, such as lowering hormone doses, decreasing fever symptoms, mitigating complications, reducing glucocorticoid and antiviral doses, stabilizing oxygen, repairing T lymphocytes and antibodies, maintaining normal liver function, and reducing inflammation (102). Two natural products, such as baicalin and baicalein, have been extracted from TCM, which inhibit the growth of SARS-CoV-2 *in vitro* (109, 110). Cinchona is a medicinal plant that produces quinine. Studies have reported that choloquinine has DNA-interlacing properties and will become an ideal candidate for developing an effective drug for treating of SARS-CoV-2 in the future. In addition, two other important alkaloids, such as palmatine and chelidonine, inhibit the replication of RNA viruses, which are considered to be an attractive antiviral drug against COVID-19 (111). Therapeutic studies on various antiviral drugs, such as oseltamivir, remdesivir, hydroxychloroquine, and azithromycin, have been conducted based on previous investigations on SARS-CoV-2. However, there is no clear evidence that antivirals can automatically minimize SARS-CoV-2 clinical reactions (81). Immunomodulatory and therapeutic effects of hydroxychloroquine against COVID-19 infection have been documented, indicating that it stimulates the growth of macrophages and reduces the risk of cytokine storms, IL-1, IL-6, and prostaglandins. *In vitro* experiments have demonstrated synergistic effects of hydroxychloroquine and azithromycin on COVID-19 (112, 113).

Remdesivir is a nucleoside analog that has been successfully used in patients with COVID-19 in China. The outcomes revealed a decrease in mortality, suggesting that it is an important potential therapeutic alternative against COVID-19. The use of recombinant human ACE2 and recombinant interferon would be a therapeutic choice for the potential treatment of COVID-19 (114).

The combination of Lopinavir, Arbidol, and Shufeng Jiedu Capsule has strengthened therapeutic barriers in patients with COVID-19 (115). The current control of COVID-19 is mainly supportive treatment (116). ARDS and cytokine storm are the leading causes of death in patients with COVID-19. However, high dose corticosteroids, especially dexamethasone and cyclosporine A, reduced or cured ARDS that severely affects lung tissues. Therefore, low-/medium-dose corticosteroids are suggested to be used in patients with COVID-19 (27). Cytokine storm accompanied by hyper-inflammation during COVID-19 is a more focused strategy and an efficient treatment pathway. Tocilizumab (Rochi®) and Anakinra have been used in managing patients with COVID-19 in China for treating cytokine waves (102). Recently, a new hypothesis for treating cytokine storms by inhibiting Janus kinases (JAK) has been suggested. These small molecules can activate the IL-6 receptor to induce inflammation. Research is underway to assess the efficacy and immunity of JAK inhibitors in severe cases of COVID-19 infection (114).

Probiotics are living microorganisms that are especially used to improve the immunity of humans. Moreover, probiotics are believed to be a possible treatment for COVID-19 by increasing immunity. However, no current clinical evidence exists for the use of probiotics during COVID-19 infection. The National Health Commission of China and the National Administration of Traditional Chinese Medicine have agreed to use probiotics in treating COVID-19 (117). Probiotic supplements help combat COVID-19 by reducing cytokine storm activation and modulating intestinal microbiota damage. It is possible that the basic principle of preventive use of probiotics in SARS-CoV2 infection is related to their ability to maintain and repair health status in the gut-associated lymphoid tissue, which prevents the entrance of the virus into intestinal cells. Large clinical trials are warranted to address these burning issues and identify the most useful and safe environment for the administration of probiotics. In fact, three registered trials have evaluated the use of probiotics, such as *Lactobacillus coryniformis*, in patients with COVID-19 (118).

Safe and efficient vaccines are required to create a protective immune response during the COVID-19 pandemic. Numerous methods have been introduced for the preparation of COVID-19 vaccines, including inactive, live attenuated, viral vector, replicated and non-replicated, Spike protein and peptide-based, and nucleic acid techniques (119). Research on the ideal candidate for SARS-CoV-2 is important for the preparation of vaccines and is underway. The ideal characteristics for COVID-19 vaccine include the following: excellent protection for healthy adults, pregnant women, babies, the elderly, and immunocompromised individuals; minimal adverse reactions, contraindications, and post-immunization non-provoking immunopathology; prompt long-lasting protective humoral and cell-mediated responses within 2 weeks using a single dose; being easily mass-produced; and being stable at 2 weeks. A total of 167 vaccine candidates are being tested worldwide. Among these, 29 are under clinical observation and 138 are under preclinical trials until 13 August 2020 (120). The first vaccine against COVID-19 was developed and approved for use only by the Chinese army (27).

COVID-19 mRNA-based vaccines are being developed, such as Moderna (NASDAQ: MRNA), chimpanzee adenovirus vector vaccine, and INO-4800, and clinical trials will soon be conducted (119). ChAdOx1 nCoV-19 vaccine has been used in treating COVID-19 positive patients and inducing protection against the disease; its efficacy and immunogenicity will be investigated in human controls. Data showed that the ChAdOx1 nCoV-19 vaccine exhibited an appropriate safety profile and improved antibody and cellular immune responses. These findings endorse a large-scale assessment of this candidate vaccine in the ongoing phase 3 trial program (120).

S protein-based vaccines are more potent than any other type. They regulate the COVID-19 pandemic in several ways, such as preventing the viral entry and attachment to ACE2 receptors and activating both humoral and cellular immune cells. They are delivered through aerosol or oral routes in individuals. INOVIO Pharmaceuticals has prepared a plasmid-based DNA vaccine targeting the SARS-CoV-2 S protein (INO-4800). This vaccine was administered intradermally. INO-4800 developed neutralizing antibodies in mice and guinea pigs with stimulating cell-mediated and humoral immune responses. Protection (94%) and tolerability were observed in phase 1 volunteers (27). Interestingly, the BCG vaccine has been shown to be safe and effective against COVID-19 in countries with a uniform BCG policy. In addition, the formalin-inactivated *Pasteurella multocida* vaccine is believed to be effective against COVID-19 during early infection. Recently, Novavax® has produced a virus-like particle (VLP) vaccine against COVID-19. The VLP vaccine can be obtained from plants and contains Matrix-M adjuvants targeting the SARS-CoV-2 S protein (121). Potential pitfalls when developing COVID-19 vaccines arise, such as structural homology between the receptor-binding domain of SARS-CoV-2 and SARS-CoV and cross-reactivity of antibodies of various coronaviruses due to antibody-dependent enhancement mechanism (122). Therefore, extra caution should be taken before making a final decision on any vaccine in the market. Additionally, the comprehensive infrastructure and financial resources needed for this project are key issues and requirements that need to be legitimately addressed. However, COVID-19 vaccines will be available in early 2021 due to emergent conditions worldwide and commercial vaccines may take 10 years until reaching final production (121).

## Interventional Effects of Various Nutrients on COVID-19

Presently, treatments or vaccines against COVID-19 are available. Nonetheless, many scientific studies have been published that highlight the general treatments for viral infections (as detailed above). Among various strategies to fight COVID-19, the use of micronutrients has shown promising results by improving immunity, particularly vitamins A, C, D, E, and B complex (especially B_2_, B_3_, B_6_, and B_12_) (123, 124).

Vitamin C has antioxidant potential, and its deficiency is traditionally linked with pneumonia. Moreover, vitamin C has been shown to have a therapeutic role during COVID-19 infection by reducing inflammatory responses, optimizing the opsonization process and the immune system, and maintaining vascular consistency (125). On March 23, 2020, Dr. Tom Frieden, former director of the Center for Disease Control and Prevention (CDC), suggested the use of vitamin D to control the outbreak of COVID-19. Several researchers identified the therapeutic effects of vitamin D on viral infections through several mechanisms, such as producing cathelicidins that have a direct antiviral effect on enveloped and naked viruses, reducing the development of pro-inflammatory Th1 cytokines, namely, TNF alpha, interferon *Y*, and IL-2, and increasing development of glutathione to regulate COVID-19 infection (126).

According to the literature, the use of vitamin B can be helpful in the early stages of COVID-19 management. Thiamine (vitamin B1) has the potential to enhance the immune system and minimize preexisting conditions such as diabetes, hypertension, kidney disease, cancer, and neurodegenerative disorders. Niacin (vitamin B3) and pyridoxine (vitamin B6) have immunomodulatory effects that limit the production of cytokines, such as IL-6 and TNF alpha, which help regulate cytokine storms in patients with COVID-19. Recently, folic acid (vitamin B9) was found to prevent SARS-CoV-2 S protein from binding to host cells. However, further research is warranted to determine if high doses of vitamin B will help in treating patients with COVID-19 (127). In a study investigating the mechanisms of action of minerals, Zhang and Liu (97) have reported that the suitable consumption of some minerals such as iron, selenium, and zinc can have effects not only on COVID-19 itself but also on coronaviruses-related symptoms, such as diarrhea and lower respiratory tract infection (128); the same results were observed for macronutrients, such as omega-3 fatty acids (129).

Numerous medical studies have reported the significant role of antioxidants in protecting lung cells against viruses and bacteria (130). Not only in coronavirus infections but also in many other infections, the balance between antioxidants (redox balance) and oxidants is altered with serious consequences (131). Viral infection leads to a surge in the intrapulmonary oxidative burden. The pathophysiological mechanisms by which free radicals lead to nitrative, oxidative, carbonyl, and endoplasmic reticulum stresses trigger lung inflammation and an altered lung immune response. In this scheme, dietary antioxidants can play a decisive role in fighting lung oxidative stress. Similarly, positive effects for the administration of probiotics and prebiotics have been reported since an imbalance in the intestinal microbiota was detected in patients with COVID-19 (132).

Nutritional interventions between related functions and the target of virus have been conducted worldwide. Moreover, the capacity for inflammatory cytokine release of nutrient-derived bioactive compounds has been demonstrated (133, 134). The release of inflammatory cytokines was suppressed by the administration of different plant-derived polyphenols to *in vitro* cultured immune cells (135). In particular, bioactive components such as flavonoids and polyphenols can act as immune modulators and inflammatory mediators and have a protective effect against lung infections. Among polyphenols, epigallocatechin 3 gallate (EGCG) is the most vigorous ingredient in green tea and exhibits antiviral, antibacterial, anticancer, antioxidative, and chemopreventive activities (136). Lately, some scientific studies have focused on the protective effects of EGCG against lung diseases such as pneumonia, chronic obstructive pulmonary disease, and asthma. Furthermore, EGCG can invade and suppress inflammation in the lungs of asthma rats. Furthermore, flavonoids may be used to lessen lung injury in mice, and they have been reported to restrain the influenza virus (129). The process to detect, prevent, and treat the disease should address the decrease in the inflammatory response and avoid the excessive postinflammatory immune cutoff, seen in such patients and defined as a compensatory response (137). Several clinical trials have shown that the curcumin spice can possibly have positive effects against COVID-19 disease via inflecting different molecular targets that play a role in the attachment and internalization of SARS-CoV-2 in many organs, including the liver, kidneys, and cardiovascular system. This spice could also modify cellular signaling pathways such as apoptosis, RNA replication, and inflammation. Moreover, it can block the combined pathways of pulmonary fibrosis and edema in COVID-19 disease. Numerous clinical trials have demonstrated that the problem regarding the curcumin bioavailability can be addressed by administering a high amount of concentration within non-toxic limits. Curcumin can be extremely efficient as an adjunct in reversing the fatal cytokine storm in dangerous cases of COVID-19. It is also interesting to report that a recent review revealed the potential impact of phytogenic plants as protective agents in both animals and humans (53, 134, 138, 139).

## Conclusions

The COVID-19 pandemic, originating in Wuhan, China, and reckless behavior worldwide have posed a significant risk to the overall population, specifically the elderly, immunocompromised individuals, and healthcare staff. So far, this virus's zoonotic spread is not confirmed; however, the phylogenetic analysis showed that bats are the primary reservoir host. Human-to-human transmission should be controlled by adopting aggressive control measures such as disinfection of living places, high standards of personal hygiene, social distancing by the community, and lockdown of the virus-prevalent areas since effective preventive strategies such as antiviral drugs and vaccinations are currently unavailable. To date, the Chinese are the only nation worldwide that have successfully recovered from COVID-19 by implementing strict HBE policies in terms of lockdown. Therefore, the WHO, CDC, and international public health authorities should continue to observe the disease status globally with rapid response systems. Consequently, the longer we face this nightmare, the better we can respond.

## Author Contributions

All authors listed have made a substantial, direct and intellectual contribution to the work, and approved it for publication.

## Conflict of Interest

The authors declare that the research was conducted in the absence of any commercial or financial relationships that could be construed as a potential conflict of interest.
